# Involvement and structure: A qualitative study of organizational change and sickness absence among women in the public sector in Sweden

**DOI:** 10.1186/1471-2458-11-318

**Published:** 2011-05-16

**Authors:** Maria Baltzer, Hugo Westerlund, Mona Backhans, Karin Melinder

**Affiliations:** 1 Stress Research Institute, Stockholm University, Stockholm, Sweden; 2 Department of Public Health Sciences, Karolinska Institutet, Stockholm, Sweden; 3 Swedish National Institute of Public Health, Östersund, Sweden

## Abstract

**Background:**

Organizational changes in modern corporate life have become increasingly common and there are indications that they often fail to achieve their ends. An earlier study of 24,036 employees showed that those who had repeatedly been exposed to large increases in staffing during 1991-1996 had an excess risk of both long-term sickness absence and hospital admission during 1997-1999, while moderate expansion appeared to be protective. The former was most salient among female public sector employees. We used qualitative interviews to explore work environment factors underlying the impact of organizational changes (moderate and large expansions in staffing) on sickness absence from an employee perspective.

**Method:**

We interviewed 21 strategically selected women from the earlier study using semi-structured telephone interviews focusing on working conditions during the organizational changes. We identified 22 themes which could explain the association between organizational changes and sickness absence. We then used Qualitative Comparative Analysis (QCA) to reduce the number of themes and discover patterns of possible causation.

**Results:**

The themes that most readily explained the outcomes were *Well Planned Process of Change *(a clear structure for involvement of the employees in the changes)*, Agent of Change *(an active role in the implementation of the changes), *Unregulated Work *(a lack of clear limits and guidelines regarding work tasks from the management and among the employees), and *Humiliating Position *(feelings of low status or of not being wanted at the workplace), which had been salient throughout the analytic process, in combination with *Multiple Contexts *(working in several teams in parallel) and *Already Ill ***(**having already had a debilitating illness at the beginning of 1991), which may indicate degree of individual exposure and vulnerability. *Well Planned Process of Change, Agent of Change *and *Multiple Contexts *are themes that were associated with low sickness absence. *Unregulated Work, Humiliating Position *and *Already Ill *were associated with high sickness absence.

**Conclusions:**

These findings suggest that promising areas for future research and improvement in change management could be the structured involvement of the employees in the planning of organizational changes, and the development of methods to avoid highly unregulated working conditions.

## Background

Studies focusing on organizational changes have indicated that there can be negative health consequences, especially in relation to downsizing [1-6], but also in relation to repeated large-scale expansion [7] and other major organizational changes [8,9]. In 2003-05, the women's share of all cases of long-term sickness absence (60 days+) in Sweden was 65%, and employees in the public sector were heavily overrepresented among those on sick leave [10]. The largest single group among those on long-term sickness absence are people working within health care, of which a substantial majority are women (84% in 2005) [11], reflecting a highly gender segregated labour market. The high levels of absenteeism in the public sector could thus be gender related, for instance due to higher levels of work-family conflicts among women [12]. However, since the sharp rise in sickness absence among women and in the public sector coincided with dramatic financial cuts [13], a major drive to increase the educational level of employees, and other changes in this sector [14], it is possible that a large part of the rise in sickness absence was due to the pattern of restructuring during the 1990s rather than gender issues per se. In addition, there are indications that economic expansion, such as that of the late 1990s, may be associated with higher mortality, partly because of increased job-related stress [15].This does not preclude that gender order could have contributed to shaping the structural changes and their health consequences, but it can be argued that it is more fruitful to look at what problems the organizations have, rather than women might have [16].

Research on work organization and management has above all embraced general trends and models of management, and not the way in which individual workers are affected by structural changes at the organizational level [13]. Brunsson and Sahlin-Andersson [17] argue that even if changes within organizations are well researched, the functional rhetoric around organizational changes gives an overly simplified picture of the phenomenon. In the social sciences, such as social anthropology, psychology and sociology, on the other hand, studies of organizations and organizational changes have investigated issues such as identity, organizational identification, globalization, and organizational culture [18] and, using qualitative (often ethnographic) methods examined organizational changes at different levels. Focus has been on the complexity of changes as well as on specific phenomena within the organizations in order to see how effective or successful the changes are, or how they are achieved [19]. An example can be seen in Saksvik et al [20] who, using interviews with managers and employees, both in the public and private sectors, have attempted to chart criteria for healthy organizational changes. They focus on the change *process*, its components and factors influencing the outcome of successful reorganizations.

From an international perspective, we can see that organizational changes nowadays tend to be implemented more rapidly than before [21] and that many of them do not yield the expected results [22,23]. Furthermore, today's organizational restructuring can mainly be explained by external pressure in terms of increased competition, more rapid pace of change, and increased complexity [24]. Since a large proportion of organizational changes fail to achieve their ends [25], there is a need to study them, in order to get an insightful understanding of the process of change [26]. When the basic structure of the organization is changed, it influences the behaviour of the employees, and may impact on their health. Employees are confronted with a new system of stressors at the workplace due to changes in the work climate [27].

A longitudinal study of 24,036 employees in Sweden [7] showed that those who had been repeatedly exposed to large expansions (increases in staffing) during 1991-1996 had an excess risk of both long-term sickness absence (odds ratio 1.07 [95% CI 1.01-1.13]) and hospital admission (1.09 [1.02-1.16]) during 1997-1999. In this context, odds ratio signifies the change in odds for each additional year of exposure, varying from 0 to 6. The strongest association between large expansion and sickness absence was in women in the public sector (1.18 [1.08-1.30]), corresponding to an odds ratio of 2.77 [1.62-4.74] between full exposure (all 6 years) and no exposure. Repeated moderate expansion, on the other hand, appeared to have a protective effect (odds ratio 0.91 [0.84-0.98], p = 0.012). These somewhat surprising results are, however, difficult to interpret, as the precise reality behind these expansions is not known. Expansion was defined according to an algorithm which used register data based on reports from the employers. It is possible that other phenomena than expansion, e.g. centralization or opening of new units not coded as new workplaces, as well as inaccurate reports from the employers, could have led to misclassification of a substantial proportion of the observed cases.

We therefore conducted the present interview study with a number of those women who according to the previous study had been exposed to workplace expansion, aiming to understand how organizational changes could affect the health of the employees. We limited the study to women employed in the public sector in order to maximize our chances to observe expansion which negatively affects health, not because the phenomena under study are necessarily confined to this group.

We hypothesized that major changes in staffing would in most cases have been accompanied by substantial changes also in work organization and work environment, although this need not always have been the case. We thus chose an open, exploratory approach, focusing on organizational changes and work environment in general, in order to better understand what the informants had been exposed to in their workplaces. In the present paper, the term 'organizational change' - which could mean a wide range of things - is therefore used to denote both planned and unplanned changes experienced by the informants, as well as changes in staffing.

## Methods

### Study sample

The informants in the present study were selected (as described below and in Additional File 1) from the persons included in the aforementioned study by Westerlund *et al*. [7], which in turn was based on representative samples of the Swedish working population 1989-1999 (the biennial Swedish Work Environment Surveys). All data in that study, and thus all selection criteria in the present study, were based on national administrative registers. We defined long-term sickness absence as medically certified absence of 90 days or more during a three-year period. The amount of sickness absence was calculated by adding the statutory number of days paid for by the employer to the number of days registered in the National Insurance database from 1997-99, inclusive. All individuals who did not meet these criteria for long-term sickness absence were defined as having low sickness absence. Change in workforce size at all workplaces in the country was computed by Statistics Sweden (by use of tax registry data) for each pair of years (November one year to November the next), adjusting for outsourcing and mergers (the Company and Workplace Demography registry). If the workplace at which a person worked in any given year had expanded by 18% or more since the previous yearly return, the individual was judged to have been exposed to a large expansion that year. Moderate expansion was defined as an increase of 8% or more, but less than 18%. Change of less than 8% was regarded as no change [7].

In the present study we chose to study female public sector employees since the association between large-scale expansions and future long-term sickness absence, as reported in the previous study, was strongest in that subgroup. We therefore selected 200 women, born between 1936 and 1956, in each of the following four groups (cf. Table 1 below and Additional File 1), from the public sector employees included in Westerlund's 2004 study [7]:

**Table 1:** *The four groups from which interviewees were selected*.

	Large expansions	Moderate expansions
High sickness absence	**I**	**IV**
Low sickness absence	**II**	**III**

• Group I, the 200 women who in that study contributed most significantly to the results indicating that large-scale expansions were associated with an excess risk of long-term sickness absence (high sickness absence).

• Group II, those who contributed most significantly to the association between moderate expansions and low risk of long-term sickness absence (low sickness absence).

In addition, we selected 'paradoxical cases':

• Group III, those who despite large-scale expansions did not become long-term sickness absent (low sickness absence).

• Group IV, those who despite moderate expansions became long-term sickness absent (high sickness absence).

This selection was done with the so-called 'jack-knife' method: We repeated the original statistical analysis of personnel change and sickness absence from Westerlund *et al*. [7] (there presented in the fourth table under the heading 'women/public sector') over and over again, excluding one subject at a time and recording how the results were affected by the exclusion of that particular person. The subjects whose exclusion affected the risk of sickness absence the most were selected.

Altogether, 800 persons thus selected were contacted by Statistics Sweden (SCB) to obtain their permission to disclose their contact information to the researchers at the Stress Research Institute. 486 persons (60.8%) responded by mail and among them 140 agreed to be interviewed (29-41 per group). We then contacted between three and seven individuals from each group for an interview. When choosing which persons to contact, we strove to select women with as different occupations, ages and geographical locations as possible within each group in order to get as rich and varied information as possible. Three of the contacted persons could not be interviewed; one had died, one suffered from rapidly progressing dementia, and one declined despite previously having agreed. Altogether, 21 interviews were conducted: seven with women from Group I; six from Group II; five from Group III; and three from Group IV (detailed information about the participants is provided in Additional File 2).

### Interviews

The contact with the informants was initiated by a letter with basic information about the study, some questions about their previous workplace as well as approval of participation. The initial letter was followed by another letter to prepare them for the interview (information about the questions etc.) and we then telephoned the informants to book time for an interview. All interviews were recorded using computer or MiniDisc technology and all the informants accepted the recording of their interviews. The interviews were transcribed in a verbatim manner by MB in Transcriber version 1.5.1 (http://trans.sourceforge.net/en/presentation.php).

The study is based on semi-structured telephone interviews, and an interview guide was used, with open-ended questions that covered a list of topics about work, work organization, and changes at work (not primarily about health). This semi-structured approach allowed the interviewer to follow leads, change the order of the questions, as well as to add and omit questions [28]. Semi-structured interviews have the advantage of 'freewheeling', typical of unstructured interviews, while the interview guide helps effectively to cover the areas of interest. The interviews were conducted on one occasion per person and ended up being relatively different from each other because of the open character of the interview and depending on what the informant chose to focus on. We asked the informants to tell us about their workplace(s) during 1991-96 in chronological order, so as to make it easier to follow their story. Of the 21 interviews, 18 were conducted by Maria Baltzer (MB) and three by Hugo Westerlund (HW) during spring 2007. The length of the interviews ranged between 25 and 50 minutes.

### The analysis

A framework approach guided the analysis of the qualitative data. This approach was developed at the National Centre for Social Research in England in the 1980s for use in applied qualitative research with relatively pragmatic aims. As a preparation we studied the area of interest by reading relevant literature and articles, i.e. the researcher was not a "blank slate". In a first analytic step we familiarized ourselves with the data by listening to the audio recordings, transcribing and reading the interviews to get a sense of the whole. In a second step we coded the text in interpretative codes, so-called *pattern-coding*, identifying a thematic framework before highlighting quotes and making comparison between cases. The text was coded with *nodes*, a labelling of the text. In the following stage we re-arranged the quotes (nodes) in a new appropriate thematic content (data reduction). All interviews were analyzed using Nvivo 2.0 (QSR International, Melbourne, Australia).

The data analysis did not take place in a linear manner; but with parts of the process sometimes overlapping each other. The analysis started at the same time as the data collection, and we went "back and forth" in the material, referring back to the original transcript throughout analysis and interpretation, and allowing later interviews to be influenced by the ongoing analysis. During the analysis, the texts were studied closely, aiming at disclosing the underlying meaning. Throughout the project, literature related to the themes in the data was studied. All interviews and themes were extensively discussed by MB and HW after independent close readings, using both the verbal transcripts and the audio recordings.

### Qualitative Comparative Analysis

Qualitative Comparative Analysis (QCA), which is suitable for the systematic synthesis of complex information about 10-30 cases, was used to minimize and structure the themes [29]. It is a method used to identify patterns, and see how different combinations of themes interact. QCA uses Boolean algebra in order to identify the minimum number of binary conditions which explain a certain outcome (here long-term sickness absence 1997-1999).

QCA was introduced by Charles Ragin and has mainly been used in political science research when the number of cases is too small for statistical analysis and too large for traditional qualitative methods [29]. The method analyses *absence *or *presence *of a certain condition - not the quantity. The method is qualitative since it builds on a qualitative judgement, and since it should be used in dialogue with the data - contradictions as well as solutions are repeatedly compared with the data, in our case the interview texts. Patterns of conditions are examined as a whole, and not separately. The cases are presented in a so-called truth table, where each row represents a pattern of absence and presence of a number of conditions, and the linked outcome.

The analysis started with a close examination of the interview texts, with the aim of identifying the presence or absence of themes. In general a one (1) indicates that the phenomenon exists and a zero (0) that it is absent for a certain informant. Regarding themes which were coded deductively, subjects can be assigned either a 1 (the theme exists for that person) or a 0 (the theme did not exist for that person) since the informant has answered a direct question. Regarding themes which were coded inductively, however, and thus not linked to a question asked of all respondents, the interpretation of a 0 is slightly different, namely either that the theme did not exist for that person, or that the theme did exist but was not mentioned in the interview.

The study was approved by the Regional Research Ethics Committee in Stockholm.

## Results

The analysis of the interviews resulted in 22 themes which covered the areas spontaneously discussed by the informants as well as responses to direct questions (Table 2).

**Table 2:** *Themes identified in the interviews and used in the Qualitative Comparative Analysis*.

**Theme**^a^	**Type**^b^	Description
Absent Manager	I	The informant described a situation where the employer/line manager was physically absent or/and unable to give support to the employees.
**Agent of Change**	D	The informant described a situation where she had an active role in the implementation of the changes.
**Already Ill**	D	Means that the informant mentioned having already had a debilitating illness at the beginning of 1991.
Control In	D	Informants described a feeling of being in control during day-to-day activities at work, e.g. regarding when and how the work tasks should be performed.
Control Over	D	Informants felt that they had substantial influence over structural issues at the workplace, e.g. the setting of new goals, organisational decisions, hiring of new employees etc.
Cost-Cutting	D	The informant expressed the view that the organisational changes were mainly driven by financial cuts.
Disintegration	D	The informant described that subgroups were formed and that former colleagues were split up making cooperation more difficult.
High Workload	D	The informant experiences a high workload with few pauses.
**Humiliating Position**	I	The informant experienced a state of low status or belittlement and a feeling of being an outsider or not being wanted at the workplace.
Insufficient Dialogue	D	The informant experienced a lack of, or insufficient, dialogue with her line manager and other superiors during the organisational changes.
Investment	D	The informant felt that the organizational changes were aimed at improvement rather than cost-cutting, and that the management invested in the organization.
Lacking Competence or Qualifications	I	The informant experienced a lack of competence or formal qualifications in relation to other employees and/or work tasks.
Lacking Exit Possibility	I	The informant had not seen a realistic possibility to find another job.
Mentor	I	The informant mentioned a person who gave support and inspiration, and who in different ways encouraged the informant during the changes.
**Multiple Roles**	I	The informant worked in several teams in parallel.
Pioneering Spirit	I	The term Pioneering Spirit (Swedish: nybyggaranda) was mentioned with enthusiasm in several interviews, a phenomenon which was described in terms of being there 'from the beginning' and 'building something new'.
Problem with Colleagues	D	The informant mentioned conflicts or malfunctioning communication at the workplace.
Stress in Private Life	D	The informant mentioned having experienced difficulties and/or stress in her social/private life.
Support from Colleagues	D	The informant experienced good support from her colleagues, both at work and in private life.
Support in Private Life	D	The informant experienced good support from her family.
**Unregulated Work**	I	The informant experienced a lack of clear limits and guidelines regarding work tasks from the management and among the employees.
**Well Planned Change Process of change**	I	The informant described a clear structure for involvement of the employees in the changes, e.g. in the form of meetings, clear information, and adequate dialogue.

^a^The themes used in the final solutions are indicated in bold.

^b^Themes coded inductively are labeled (I) and the ones coded deductively are labeled (D).

### Minimizing the themes using Tosmana

In a first step, we ranked the 22 themes based on our knowledge of the data in such a way that the themes which we believed had influenced the outcome the most were on top. In order to minimize the themes with QCA we followed a checklist as proposed by Rihoux and Ragin [30]. We started by analyzing the 10 most highly ranked themes/conditions using Tosmana (Indicated in bold in Table 2), a software constructed for QCA [31].

We conducted separate analyses for the outcome "high sickness absence" and the outcome "low sickness absence". When logical remainders, i.e. theoretically possible but non-existing cases, were included and treated as if they did not contradict existing cases, we were left with one solution for the outcome "high sickness absence" and four alternative solutions for "low sickness absence" (see Table 3). We also ran the analyses excluding logical remainders but Tosmana failed to reach a minimal solution. We also tried entering only five conditions at a time, as one may argue that 10 conditions are too many for 21 cases. This exercise gave the same result as when entering 10 at the same time, but the fourth solution for "low sickness absence" was dropped.

**Table 3:** The conditions that were extracted by QCA in order to get a simplified pattern.

Combination of conditions logically explaining	Combination of conditions logically explaining
HIGH SICKNESS ABSENCE	LOW SICKNESS ABSENCE
Unregulated Work	Agent of Change
OR	OR
NOT Well Planned Process of Change AND Already Ill	Multiple Contexts AND NOT Unregulated Work
OR	OR
NOT Multiple Contexts AND Humiliating Position	NOT Unregulated Work AND NOT Humiliating Position AND NOT Already Ill

In order to test inter-rater reliability, we asked two people with experience in coding qualitative interviews to judge the presence or absence of the six themes used in our final solution (cf. below) in four (different) interviews each (one interview each randomly selected from each group in Table 1). Each rater thus judged 4 × 6 binary conditions, e.g. if the interview with Monica showed that she had been exposed to a Humiliating Position (1) or not (0), based on the interview texts and the definitions of the themes provided in Table 2. The instructions to the first rater were, unfortunately, incomplete, which is why she used a question mark when she was uncertain. Since we had chosen to code uncertain cases as 0, question marks should presumably be interpreted as 0, and the second external rater was explicitly told to use 0 when uncertain. Rater 1 was in full agreement with us 15 times (62%; counting one occurrence of '0?' as '0'), in questionable agreement six times (25%; i.e. had '1?' or '?' when we had '0'), and disagreed three times (12%). Rater 2 was in full agreement 23 times (96%) and disagreed once (4%). We calculated Cohen's kappa, which was 0.72 if questionable agreement was interpreted as disagreement, and 0.78 if interpreted as agreement. In line with Landis & Koch [32] we understand this as substantial agreement, and thus good inter-rater reliability.

### Combinations of conditions

Table 4 presents a so-called truth table with all interviewees. As can be seen, the presented subset of six out of 22 conditions is unambiguously related to the outcome (long-term sickness absence) among the respondents. This subset of six conditions corresponds to the final solution chosen in QCA, and represents the smallest subset of conditions that can explain both presence and absence of the outcome.

**Table 4:** *Truth table, i.e. different combinations of presence (1) or absence (0) of conditions, and an outcome indication*.

Informant/Case	Outcome (sickness absence)	Multiple Contexts	Agent of Change	Unregulated Work	Well Planned Process of Change	Humiliating Position	Already Ill
Sofia	1	0	0	1	0	1	0
Monica+ Barbro	1	0	0	0	0	0	1
Sara	1	0	0	1	0	0	0
Maria	1	0	0	0	1	1	0
Maud	1	1	0	1	0	1	0
Märy	1	0	0	0	0	1	1
Anna + Tuulia	1	0	0	0	0	1	0
Mona	1	0	0	1	0	1	0
Carolina + Eva	0	0	0	0	0	0	0
Kerstin + Maggan	0	0	1	0	1	0	0
Inga	0	1	0	0	1	0	1
Olga	0	1	0	0	0	0	0
Birgitta	0	1	0	0	0	1	0
Gunilla	0	0	1	0	1	0	1
Gertrud	0	1	0	0	1	0	0
Ann-Sofi	0	0	0	0	1	0	0
Birgit	0	1	0	0	0	0	0

N.B.: Logically possible combinations that do not correspond to actual cases are not displayed.

The final solutions (Table 3) encompass the combinations of conditions that in the most economical way explain the outcome "high sickness absence" and "low sickness absence" for the 21 interviewees. Among the four possible solutions for "low sickness absence", we chose to use the one which contained only conditions that were relevant in all four solutions. This solution was also seen as theoretically more interesting since it included components that have not been studied extensively before.

The reason why we thought that certain themes were important was partly that the phenomena were already well-known in the literature as factors influencing health, and partly that several of our interviewees had mentioned these themes several times and/or emphatically. When analyzing the material in Tosmana, we saw that, according to the informants' stories, some themes which we had thought were important could not by themselves explain either of the outcomes "high sickness absence" or "low sickness absence". Themes not included in the final solutions may indeed still be important, but in our material they were logically neither sufficient nor necessary to explain the outcome in the chosen solutions, which indicates that other factors may be more crucial.

Below we discuss and describe the meaning of the six themes in the final solutions in more detail.

## Themes in the final solution

### Unregulated Work

Unregulated Work means that the informant describes a work environment where there are no clear limits or guidelines regarding work load or work tasks. There is a lack of daily routines and well formulated work assignments and the informants describe a feeling of being left to solve new situations and problems all by themselves. We believe that it describes a contemporary phenomenon like more flexible structures within organizations and new demands in working life [18]. New types of relations between employees and the organization develop, and more flexible organizations are in demand [33]. This could be an opportunity for the employees to 'grow', to increase the freedom in their work. However, our interviews indicate that this increase in flexibility could also create ambivalence, where the informants described how they didn't get any, or only insufficient, information.

The informants also described experiencing lack of time and competence needed to solve problematic situations in their (new) positions. The informants were not prepared for their new roles or positions in the organization. Working hours increased because of too many new responsibilities, problems and work tasks. The informants worked evenings, week-ends, and/or during lunch hours to get time to handle their new work tasks and the problems related to these. These circumstances are illustrated by Jimmieson *et al*. [27] who argue that the employees could experience 'role overload' during organizational change, that is to say too many assignments arising which exceed the employees' skills and capacities.

The informant below describes how she and her colleagues had to solve new problems and situations at the workplace by themselves, and how, lacking in clear boundaries, her work situation consisted of several very vaguely described work tasks that could not always be foreseen.

And then we were you know, um... And then we came to the point where it like, what wasn't forbidden, you know, everything that wasn't expressly forbidden was permissible. It was all very new to us. And sometimes it went all wrong, certain things, and sometimes it turned out very well.

I was there to serve everyone. And it was you know, like I was saying, when things were going well and I was in a good mood, I said I was the funnel and when things weren't going so well, then I was like the trash can. It was everything from "Sara, there is no toilet paper" to "Now we're late with the Quarterly Financial Statement".

Nope, there were no limits. It was just "ask Sara". And that... that wore me out enormously.

(***Sara, finance clerk***)

Jimmieson *et al*. [27] also point out that role conflicts are probably more frequent during organizational changes since the expectations of the new organizations can be the opposite of those of the old ones. The informants feel that it is unclear what their new roles mean and what tasks and positions they involve. Obstacles such as unclear goals and assignments or a lack of resources are typical in this situation, as well as time pressure, poor monitoring of working time, and conflicting demands, according to Härenstam *et al*. [34].

It seems that involvement increases the feeling of ownership in the changes [22]. Kerber *et al*. [35] argue that there is a general conviction that being involved and taking part in the process is a preferred strategy in organizational change in order to avoid negative reactions (cf. Agents of Change below). At the same time it could be dangerous to use such a strategy if the involvement gets too intense, or on the contrary, does not contribute to anything creative. If the involvement is executed without boundaries or instructions, it could lead to a situation where the group or work team becomes too strong and takes over, leading to group think which stifles creativity and silences critical voices. A home-help service manager describes how the work had no boundaries, which led to an increased work load as well as work pace:

And then, well like in the beginning it was like you know, whatever the suggestion was, we kind of cried

Out 'Yeah, great fun!', (laughter). But, now afterwards, well it *was *fun but it was..., I probably would've done it in a different way. (...) Regarding the working pace maybe and I don't know...

Well, it's tough to start something up, you know, it's kind of tough. And if you get paired up with a real go-getter, you know, and if you're a driver yourself, then it's ... you know, there's nobody to set boundaries for you.

(***Sofia, home-help service manager***)

Involvement is thus not a solution *per se *but has to be implemented and carried out in the right way [35].

According to Reich [36], the new, 'ideal' type of organization gives priority to identifying and solving problems. New types of relations between employees and the organization develop, and more flexible organizations are in demand [33]. This could be an opportunity for the employees to 'grow', to increase the freedom in their work. However, our interviews indicate that this increase in flexibility could also create uncertainty regarding how to perform the work and what is expected; and the informants described how they didn't get any, or only insufficient, information in these areas. In the literature it has been argued that organizational changes involving increased autonomy and flexibility are usually a means to increase productivity, and that the end result is intensified work and higher demands [37].

Unregulated Work is one of the most complex themes (i.e. a theme comprising many related codes) that emerged, and in QCA, Unregulated Work was by itself a sufficient condition to explain sickness absence in our data. This indicates that Unregulated Work can be a serious health risk. This theme comprises many aspects that in previous studies have in their own right been associated with stress and ill-health. But although most components of the theme are well-known, the originality in this theme lies in its composition.

Considering the association between the presence of Unregulated Work and sickness absence, it is hardly surprising that the absence of the same theme has a protective effect. Absence of Unregulated Work together with the presence of Multiple Contexts, where the individual is alternating between different workplaces and/or work teams, appears to create a situation where the individual is relatively protected during the change. Multiple Contexts can give perspective on the organizational changes and whatever happens around them in terms of social conflicts, new unregulated work tasks and times, etc. and could probably influence the identification with the workplace, which should decrease in strength.

### Well Planned Process of Change

In the theme Well Planned Process of Change, the informant has seen, described and benefited from a consciously created structure and active involvement of the employees during the change. This is exemplified by meetings which provide appropriate information and interactive commitment. In the interviews the informants describe procedures, access to information, and extent of involvement in the changes; in short, how the change came about in terms of communication between different parts in the organization, and if there was enough time to carry out the changes. The informants felt that they were able to follow the changes, and that they, in good time, had a chance to get used to the idea of the change both in theory and practice. This theme complements and expands the general discussion about information in organizational change; here it is a question of creating a structure for using the experience of the employees and creating involvement, time and engagement in the process.

Changes have been shown to yield better results if the employees are prepared to change their own patterns of behaviour, attitudes, goals and values [38-40]. The theme Well Planned Process of Change encompasses positive experiences of change, where the needs of the employees are considered and where there is enough time and space for the changes to develop in a constructive way.

Absence of a Well Planned Process of Change, on the other hand, means that the situation could have been more or less 'turbulent' as described by a couple of our informants. It can also denote a less chaotic situation, where the change process - particularly regarding involvement of the employees - was simply not well planned for in advance. Together with pre-existing health problems, lack of structure and involvement in the change process seems to have been the final straw for some informants: They could not deal with the strain brought on by the organizational change given that they already had a vulnerable health condition.

A lack of Well Planned Process of Change is briefly described by the informant below:

Maria: And it went pretty fast this change? It wasn't anything you spent time on talking about or tried to prepare was it?

Mona: No there wasn't much preparing, maybe the spring term a bit, or the spring term -94.

(***Mona, childminder*)**

In the organizational and management literature, as well as in public opinion in general, there tends to be a negative image of how employees in the public sector perceive changes - and that this is the result of individuals feeling insecure in general. According to Huzell [24], however, it is rather a question of the employees feeling insecurity about *how *the work should be done or *in what way *the organization will change.

When there appears to be a lack of a structured change process or the informant is not sure of what the intended organizational result is, the change tends to be described as turbulent:

Yes, then there was such a turbulence, a lot of things happened, people were moved around and functions were moved around here, there and everywhere (***Sara, finance clerk***)

That was a process that was very, very turbulent. (***Mona, childminder*)**

van Knippenberg [26] points out that successful change is often a result of how the communication in the organization works: how the employees are informed about the changes, the quality of the dialogue between manager and employee, and if there are strategic meetings. Ashford emphasizes in the following quote the importance of adequate information and dialogue: "During organizational transitions, two types of information seem most relevant: information about the change itself and 'feedback'" [41]. The informants below give us a picture of two different situations, one that exemplifies good communication (the first one) and one that exemplifies the lack of a dialogue about the changes (the second one).

Gunilla: Yes, we participated very much in that, but if it was a small group or if it was the whole group, that I can't say. But we participated very much, because I know that we had to do ... how it was going to look like and that sort of thing and, yes.

(***Gunilla, nurse assistant***)

Birgitta: And then I have the feeling that it kind of started to become like this, like when we protested, when we started to send questions to the management, then they were always sent right back: what are *you *doing?

Maria: OK, so there wasn't really a functioning dialogue then between those who managed the changes and the rest of the personnel?

Birgitta: Yes, I know that this manager said that "well you weren't born yesterday were you?", that kind of thing was what you heard when you asked a question.

(Birgitta, social worker)

Information that the employees only get through rumours and gossip has been shown to make the employees feel insecure and worried [42]. The informant below gives a good example of what it can be like when the employees get information through gossip and how this leads to insecurity at the workplace due to lack of proper information.

Maria: How did you get the information about things being, about these changes? Or the downsizing, or what... were you fewer or what?

Tuulia: Well, actually there wasn't really any information, it just happened. More or less.

Maria: Were people worried before, were there rumours about what was going on, or anything like that?

Tuulia: Yes. They were.

Maria: So you can't..., there wasn't really a functioning dialogue between those who managed the changes and the rest, no...?

Tuulia: No. It wasn't like that.

(***Tuulia, office employee, assistant clerk***)

Maria: Was that something you also had a meeting about or anything like that? How ...

Tuulia: No, not really, on the contrary it all happened a bit in secret. Well, so that was I guess what I thought was a bit of a shame. You weren't informed but there are rumours instead and that's not good you know.

Maria: No, of course. So first there were rumours started and then at the end you ... ...

Tuulia: ... found out, then

Maria: How was that then, was it the manager who came out and informed?

Tuulia: Yes...

Maria: But then there wasn't really any discussion, but it was more of a ...

Tuulia: Yes, it was you know, all decided, so to speak.

(***Tuulia, office employee, assistant clerk***)

Insecurity about the future and insufficient information during organizational changes can influence the results [27]. In order to avoid lack of information it is important to provide, informal and formal, timely and accurate, information [41].

"Maggan" describes a situation where there was a continuously functioning dialogue between the manager and the employees:

Maggan: We had one person on the staff who was absolutely fantastic, she was really good at all the conditions and regulations and things like that so well she could really use the system to the full for everybody.

*Maria*: So there was some security, you knew what happened after all and you had somebody to talk to and ask ...

Maggan: Yeah sure, all the time. And we were you know, we were you know very frank and like that, yes. I know I had, I consulted (*name*) to talk about different cultures and cultural clashes and things like that. There were a lot of things like that.

(***Maggan, personnel manager***)

If the informant didn't have a chance to benefit from a Well Planned Process of Change and also suffered from being Already Ill, she was exposed to an unhealthy working situation with a turbulent environment and vulnerable health.

Well Planned Process of Change, refers specifically to how the change process was managed. The theme Unregulated Work, on the contrary, describes a situation with a lack of leadership and/or lack of clear tasks and work areas, and even though this situation may be present during change, it is not the change itself that is the problem. It is thus quite possible to have a well regulated work and still a poorly planned process of change, or vice versa, even though it is likely that good structure in different areas often go together.

### Already Ill

This theme covers cases where the informant had mentioned already having a debilitating illness before and/or during 1991-1996, and where the outcome could readily be explained by pre-existing illness and thus not as an effect of the changes.

Maria: Um ... how was your health do you think during this time?

Inga: My health has been up and down a bit because actually it was, the 90s were the worst ten years in my life. (laughter) Yes, you know it's like this, I have lived. I had twins when I was forty-two and I was a single parent. And during the 90s I also had a father who was, who started to get sick absence and he became mentally changed and in the end passed away in '98, but these years, then there were both the children, first you know I had an intensive job. My boys went to school and were active in sports and there were lifts to be provided here and there during evenings and weekends, and then I also had my old father who needed me incessantly and day and night ... ... so you know I was falling apart some of the time, and I had a slipped disc, gallstone, thyroid gland, completely failing so, had to start with high doses of Levaxine.

(***Inga, registered nurse***)

Being Already Ill makes the informant more receptive to complicated and stressful situations such as a lack of Well Planned Process of Change. Not having this disadvantage would not solve the situation, but in combination with the absence of Unregulated Work and absence of Humiliation Position, it indicates a protective environment for the informant.

### Multiple Contexts

Multiple Contexts denotes that the informant is a member of more than one relatively independent social constellation or team in her work. The informant takes part in these groups or teams with clear boundaries between them. This can create an opportunity for the employee to get some breathing space and distance to the ongoing changes. The informants describe this as standing outside of the changes and not being totally engrossed in them.

Having Multiple Contexts can thus help to create a break from a dysfunctional working situation. As pointed out before, role conflict, role ambiguity, and role overload occur in situations with new positions and work tasks. But if the informant has two or more contexts and a chance to 'take a break' from the change process (with possible social conflict, lacking structure, stress, etc.) it can be a relief from a stressful situation.

A specialized nurse with a strategic role describes that the changes did not affect her directly; she felt that she "was standing on the side":

This is all I know. As for the rest I didn't take part personally in those changes. I kind of had my own job on the side, so to speak... (***Inga, registered nurse***)

An employee in the social services explains how she found her own 'specialty' and worked at different places:

I tried to go ahead with it you know, so that we would have a network - that sort of thing. I thought it was pretty poorly received by the others, so I started a lot of different things. And I travelled around and gave lectures on [...] and things like that. So you know, I worked pretty actively on that and sort of found my own niche there.

And actually, you know I had it pretty good. I guess I'm the type of person who needs a free rein and I had that with the refugees. I was kind of left to my own devices.

(***Birgitta, social worker***)

The fact that Multiple Contexts in the solutions appears in combination with absence of Unregulated Work or together with Humiliating Position, suggests that the main function of Multiple Contexts in relation to health is to help the employee to put things into perspective and get a "pause" from a demanding situation.

### Humiliating Position

Humiliating Position is described by the informants as something problematic and burdensome, a feeling of not being in control, of having no influence, and not being seen and heard. Unless the individual has Multiple Contexts, which seem to be protective, Humiliating Position leads to a trying situation where the informant's health may be at risk.

It was difficult to find a correct name for this theme, since it covers both a *position*, e.g. when an employee's competence is questioned leading to a more or less persistent feeling of low status and/or belittlement, and a *situation *where the employee experiences feelings of being violated and/or excluded, e.g. when the employee is expressly told that she is unwelcome at her workplace. Other examples of Humiliating Position from the data are that the interviewee was demoted to less advanced work tasks, that her competence was questioned, or that she feels that she had been fired without a 'proper' or even understandable cause.

Maria: I see, OK. But it was like, would you call it some sort of parting or how ...

Märy: Yes, we were given notice to quit from that care centre. Well, all of us who were over fifty. I think it was four of us. On the, yes the whole clinic then. At least four. I don't remember if it was five, but we were four, that I know.

Maria: Yes, so what happened next? Then you changed, you looked for a new job then or what?

Märy: Yes, I wasn't out of work. Instead they suggested that we started at the hospital. And then I said I didn't want to. But yes, I got you know a temporary job ... excuse me ... (starting to cry)

Maria: But does that feel tough?

Märy: I got different temporary jobs, then ... because I wanted to be a district nurse. And they didn't let me do that.

Maria: Are you feeling OK or do you think that it is tough for you to ...

Märy: It was really tough then. Really tough. I had, you know, been at the County Council for so many years, couldn't imagine this situation.

Maria: No, of course, it must have been a chock, mustn't it?...

Märy: Yes, you could say that. Absolutely.

Maria: But the hospital, that was something else.

Märy: No, that was you know just like, we were, you know, kind of, another world and a different job, so that was something we'd gotten away from. And I'm saying I quit at the end of the 60s, and I only had one or another temporary job after that.

(***Märy***, ***district nurse***)

Jimmieson *et al*. [27] argues that employees who doubt their ability to live up to the demands that are put on them in the new organization run a substantial risk of giving too much attention to feelings of insufficiency and incompetence, which makes it difficult for them to handle the situation. Below, an informant describes a situation where her competence was questioned by the headmaster in the school where she worked:

Now maybe I'm being a bit catty towards the principal here, but I complained about this and she said that, well, it was simply because I wasn't competent enough. Because I'm only qualified as a childminder. Yes, (laughter) ... so that's just how it was, 'cause you know most of the others were professionally trained recreational pedagogues. Or recreational leaders.

(***Mona***, ***childminder***)

There is reason to believe that Humiliating Position may arise particularly during organizational changes. In our data, several informants describe having been subjected to a Humiliating Position during, or in connection with a re-organization. During organizational changes there is a risk that important attributes, qualities and assets are lost, e.g. power, prestige or the feeling of cohesion and fellowship at work, since organizational changes often imply new roles and functions for the employees. A Humiliation Position can occur when employees, without much forewarning, either lose their jobs or experience a series of changes where they may first get a promotion, and then later be forced to take a demotion to their previous levels or below.

Maria: Were there other social relationships with colleagues that came to an end or changed as well?

Tuulia: It did, a bit.

Maria: Was there any situation you can, you remember?

Tuulia: No, not exactly, it was more that you got the feeling ...well just that, some things weren't for me to decide any more you know.

(*Tuulia, office employee, assistant clerk*)

Employees who feel loyalty towards their organization and what it represents find it easier to accept changes [43] and it is therefore easier for them to view the whole process of change in a positive light. On the other hand, the informants that suddenly realized that they no longer had a place in an organization they had previously strongly identified with, and been loyal to, described this as an almost traumatic experience.

The identification with the organization can change radically as a result of an organizational change and lead to disappointment with the new 'ideology' [27]. Organizational identification could be defined as "The perception of oneness with or belongingness to an organization, where the individual defines him or herself in terms of the organization(s) in which he or she is a member" [44].

Maria: Do you think that there was, did actually any change occur for you practically?

Birgitta: Well it actually didn't you know, cause I'm stubborn you see and I do it my way. But it causes inner conflicts, and then I'm in a context that doesn't really accept me, you see.

(***Birgitta, social worker***)

Below, a situation is described where the informant felt uncertain, inadequate and perhaps under stress in her new work role, a problem which she felt was ignored when she talked about it with her boss:

Cause I kind of... the first time, yes well before every new class, it was really hard for me to go in. And then, then I guess after the first ten minutes, things settled down. And that's how it was most of the time, but I later realized that I had been walking around terrified that they were going to find out, that they were going to really put me on the carpet, you know. Just ask me questions that I really didn't have answers for, that sort of thing.

Yeah, I did pretty much [discuss the situation]. And especially with my boss, because, you know I wanted to have fewer teaching hours. And regarding that, well, we had lots of discussions, so..., and even ...some were even pretty aggressive discussions you know and I was... or really distressed and I got really mad and said "I don't want to, I can't", and she says "I know you can, you're good at this, I know that you know how to" and "I want you to have this training". And even though I would say no twenty times, she knew if she asked long enough, I'd say "All right then".

(***Sofia, home-help service manager***)

Lack of feelings of unity and belonging among employees who are unprepared or do not identify with the new organization can be an obstacle to successful change [45]. The expectations on the employees from the new organization can be of a totally different kind than those from the old one, which can lead to insecurity and a feeling of no longer belonging. It may also be important that the discrepancy between the philosophy of work expected by the organization, and how things are done in practice, does not become too great [46].

The results of our study indicate that Humiliation Position can be particularly dangerous when the informant is "locked" in the working position with no opportunity to rest from a stressful situation as when not having Multiple Contexts. Not having Humiliation Position at work can be protective if combined with absence of both Unregulated Work and Already Ill.

### Agent of Change

Agent of Change in our material denotes that the informant had a pronounced role in the implementation of the changes, or describes that she was in some way leading a collective process which gave influence over the change. The informants described their roles during the change, whether they had been more active or more passive, if they had a clear and structured function as a driving force during the change, and if they felt they could influence decisions and processes; if the informants had time to reflect on what was happening and why it was happening. An active role or function contributes to a more obvious participation during the changes, the informants interact with others in meetings etc., get adequate information about what is happening, and feel, to a greater or lesser extent, that they are handling the changes well.

An Agent of Change is usually already from the beginning positive towards the changes, and is also allowed to be part of a process characterized by participation. She is usually also a person in a managerial or supervisory position and with a certain level of status within the organization. Those informants who described that they had been Agents of Change expressed that they had a calm and accepting attitude during the reorganization.

As already mentioned, the management literature gives the impression that among both theorists and practitioners there are many who are convinced that involvement of the employees is the best way to avoid problems during organizational changes [22]. If an employee is a driving force in addition, she has many advantages like being informed in advance, something which Sutton and Kahn [47] and others describe as predictability and understanding of the situation. With substantial likelihood, this type of situation can facilitate the employees' adaptation to the changes [47] and should decrease the negative effects of change-related stress. For instance, low involvement was shown to have negative effects on 'role stress' [48,49].

An employee in a care unit described how she led groups during the organizational change:

Well, we had a training-course, ... it was called (*Name*) ...I (...) was in charge of the study circle. And it was great. It was all about collaboration and ... how you want to develop and ... and what you, you know, the purpose was also what kind of thoughts you had when you were younger, what did you want to become?

Yeah well ... Sure, it was this (*Name*), you know that was before we started the service flats. Cause I was always planning for the future. And there was a lot of, you know, brainstorming. This is the kind of thing the staff got.

(***Kerstin, group-leader of a care institution***)

An employee in the social services gave an example of how she actively worked to implement the changes:

And we, you know we were also building up something new, cause you know, see it was this thing about functional disorder and in that, there's a lot of information there, that was transferred to [...], and this made these working teams..., they came to grow during a period of time and I was one of those who was in on building this group.

(***Birgit, social worker***)

One informant in a managerial position described how her work included implementing the changes:

Yeah, well there were a lot of meetings and discussions and things about what they should be doing out there in the districts - concrete and practical things. And we tried to find ways, accessible ways, you know, for them to get reasonably logical instructions.

So in the management groups and with the department management, this got discussed nearly all the time (laughter). How were we going to do this? How were we going to help them and ...

(***Maggan, personnel manager***)

Many studies indicate that stress increases during organizational changes [50,51] which could lead to negative attitudes towards, and during, the organizational changes [27]. Constructive change-oriented behaviours have become a responsibility or demand in the 'new employment relationship' [52] which could contribute to better conditions for the employees during an organizational change.

Agent of Change did not have to be combined with any other theme in the QCA solutions; the presence of it was enough to protect the informant from an unhealthy work situation.

### Other themes

More surprising than which themes were included in the QCA solutions was the absence in the final solutions of some themes that we thought would be of importance (some themes seemed to be of less importance for the outcome than we had thought): Social Support was one of them, an assumption we had made based on the literature about work and social support and its effects on stress and health. Disintegration was another theme we thought would be important: it illustrates a situation where a change of structure in the organization leads to disruption in work teams or in the cooperation between colleagues. The theme Pioneering Spirit (Swedish 'nybyggaranda') emerged as an interesting code, named after the word that the informants most frequently used. Pioneering Spirit was often mentioned in relation to High Work Load, strong enthusiasm among employees and colleagues, 'freedom and responsibility,' presence of group think, and Unregulated Work. Most of the informants who mentioned Pioneering Spirit in the interviews got sick-listed. High Work Load was not included in the solutions, maybe because it was masked by Unregulated Work. The lack of inclusion of Lacking Competence or Qualifications can have a similar explanation; it is often mentioned in connection with Humiliating Position.

Even though some informants mentioned work-family conflict, not many did, and we did not specifically ask about such information in the interviews. Unpaid work is often an important factor [53], but nothing we noticed specifically in our material.

## Discussion

This study explored the relationship between an occupational environment of structural organizational changes on the one hand and sickness absence on the other hand, and what the underlying factors are. Semi-structured interviews were held with 21 female public sector employees working in organizations that had expanded in the previous year.

Among our informants, Unregulated Work was logically sufficient to explain future sickness absence. The informants who had experienced this described a combination of high involvement and a severe lack of structure which in the end became more than they could manage. Those who had been Agents of Change, on the other hand, had been able to weather the changes well, largely because they were well informed and had substantial influence over the changes, but perhaps also because they had been selected for their openness to change.

Lacking Multiple Contexts, a person exposed to a Humiliation Position has few possibilities to withdraw and recuperate from a difficult situation. On the other hand Multiple Contexts is not sufficient to "handle" a change, but in the absence of Unregulated Work (which probably means a somewhat stable working situation) Multiple Contexts may create enough leeway to protect the employee during the organizational changes. For those who were not Already Ill, absence of both Unregulated Work and a Humiliating Position seems to protect against turbulent structures and individual stress, making the informants able to handle the change well. But without a stable base, like a Well Planned Process of Change, the organizational changes seem to be the "last straw" for those who were Already Ill.

Of the six themes that were used in the final solution in Table 3, two refer to characteristics more of the individual than of the work environment at the workplace where the changes are happening, namely Already Ill and Multiple Contexts. Thus, these two themes cannot easily be used to guide future change management at the workplace level. Four themes remain. Two of these, namely Well Planned Process of Change and Unregulated Work, refer to structure, albeit in different contexts. The remaining two themes, Agent of Change and Humiliating Position, imply active involvement and exclusion, respectively. It may therefore be fair to say that our study indicates that two important aspects of change management may be a clear structure and active involvement of the employees.

### Strengths and weaknesses of the study

A major strength of the study is that we identified interviewees based on a previous epidemiological study [7]. Thus we were able to select not only those who had contributed the most to the results in the earlier study, but also 'paradoxical cases' who could provide counter-evidence. Together with the evidence from quantitative data, our study provides indications regarding the potential harmful effects of ill-planned organizational changes. As with all studies based on a small number of interviews from a selected population, the results of our study must be interpreted with caution. Both the 22 themes and the minimization through QCA are based on the 21 interviews, and cannot necessarily be generalized outside of this group. Instead, the solutions presented should be viewed as a way to identify and explain social processes that have generalizing effects [54] or as candidates to be tested in future studies.

The interviews were performed 11 to 16 years after the exposure to the changes, and also long after the supposed outcomes had occurred, which may have led to recall bias and loss of detail. However, interviewing the informants so long after may also have created a certain distance to the events making it easier for the interviewees to tell their stories in a structured way, and not focus too much on emotions. We believe the stories were told with enough richness in details to be essentially trustworthy.

In order to be able to detect new phenomena related to organizational changes, we decided on a basic, explorative approach, focusing on the informants' narratives regarding the changes and their impact on the work environment while actively trying to disregard any presumptions concerning for example class, gender, or ethnicity. While increasing the chances of detecting new phenomena, this approach also limits the possibilities to analyze the material from specific theoretic perspectives because there may not be enough information in certain areas, e.g. on gender relations. We did not include men in the study since the association between major expansion and sick leave was non-significant among men in Westerlund's [7] previous study, likely because of insufficient power.

As explained in the introduction, we chose not to use a gender lens in our analysis. It can be argued that many or even all of our themes could reflect a gendered reality. Humiliating position could for instance be objectively more common among women due to structural subordination of women, and women could also be more likely to blame themselves and psychologically internalize organizational problems [55]. However, as the design of our study does not allow us to draw any empirically based conclusions regarding structural influences or differences between men and women, we have refrained from more detailed discussions about gender.

QCA was used as an analytic aid, and although the method itself builds on formal Boolean algebra, it is essentially a qualitative tool, which helps the researcher to see complex relationships between the themes which have emerged in the qualitative analysis. Because of its logical nature, it can help the researcher avoid conclusions which are contradicted or unsupported in the data. The final choice of particular solutions (combinations of themes which logically explain the studied outcome in the material), however, is once again dependent on qualitative judgement, as are the parameters that are entered in QCA in the first place. The main danger of QCA is probably that the seemingly quantitative nature of the analysis leads the researcher or the reader to over-interpret the solutions and see them as general causal models, which they are not.

The choice of one solution for Low Sickness Absence, from four possible ones, may constitute another weakness. However, since all solutions were relatively similar, and since our choice was based on intimate knowledge of the data, we believe that the selected solution reflects the patterns in the data well.

### Discussion in relation to other research

Modern reorganizations may be qualitatively different from those of earlier times. Whereas Lewin [56] described organizational changes as progressing from unfreeze via moving to freeze; modern theorists describe more complex processes [57]. Paulsen *et al*.[58], for example, describe the stages pre-structuring/pre-announcement, execution of restructuring and post restructuring, which often also tend to remain in a more uncertain state and may never reach the refreeze stage [59].

We believe that these more complex, continuous changes mean that there are new, more complex needs during changes today. Thus the goal of this study was not to find one single cause of sickness absence but to look for common features during organizational changes and identify situations where the individual finds herself in a vulnerable position *or *in a protective scenario. In this study we found some common themes that appeared to have contributed to a negative or positive outcome for our informants after reorganization. Awareness of such risks may facilitate the management of organizational change.

Considering that not only downsizing, but also expansions and other forms of organizational instability [7,8] could lead to sickness absence, it is important to understand what constitutes a good, as well as a bad, organizational change in terms of health. The studies that inspired us were above all those that give a clear picture of typical procedures and processes during organizational changes, what the consequences of these are, and how they can be understood [60]. Specifically, it can be argued that the focus should be on *what *happens and *how*, which, according to Murphy [61], is the essence of the understanding of change: *What *the change in fact influences, e.g. working hours, and *how *it affects procedures, acting, involvement etc [61,62].

The combinations that we saw when using QCA were not surprising. Well Planned Process of Change, Humiliating Position, Agent of Change, and Unregulated Work were themes that had been salient all along the process of analysis and combined with less complex themes such as Multiple Contexts and Already Ill show us a pattern where the individual is in a vulnerable position. Also it is not surprising since earlier studies tell us that poorly organized changes could be negative both for the organizational outcome and for employee health. The study gives an indication of factors that could be of importance for the sickness absence in (major) organizational changes.

## Conclusion

In summary it appears that two key factors in healthy organizational changes are active involvement of the employees and a good structure which can balance the uncertainty of the changes. Involvement can be both individual, exemplified by those interviewees who had themselves been Agents of Change, and collective, as in the Well Planned Process of Change. Structure is a key feature of the Well Planned Process of Change, while lack of structure characterizes Unregulated Work, although in that case in relation to the daily work tasks rather than the change *per se*.

From an individual perspective, having Multiple Contexts may decrease the exposure to the negative aspects of a sub-optimally managed organizational change, while being Already Ill, hardly surprisingly, may increase vulnerability. Of particular note is that employees who find themselves in a humiliating position during the changes - an apparently not uncommon occurrence - are at particular risk. Treating employees with respect, consideration, and dignity is therefore particularly important.

Knowledge about good change management and awareness about situations that create vulnerability could be used to improve future workplace interventions in order to protect and promote the health of the employees. It would also be possible and desirable that the results are used as a base for quantitative studies such as the construction of questionnaires in order to test the generalizability of the findings. Future studies should also elucidate if gender determines how organizational changes influence the employees, and/or if men are similarly affected.

## Abbreviations

***Tosmana***: a Tool for Small-n Analysis, used to perform social science research on data sets with a small number of cases; ***QCA***: Qualitative Comparative Analysis is a new analytic technique that uses Boolean algebra to implement principles of comparison used by scholars engaged in the qualitative study of macro social phenomena.

## Competing interests

The authors declare that they have no competing interests.

## Authors' contributions

HW designed the study. MB and HW made the interviews. MB transcribed the interviews, analysed them, and wrote the first and successive drafts of the paper in dialogue with HW. MoB performed QCA together with MB and HW. KM advised the authors on QCA. All authors read and revised the successive versions of the manuscript.

## Author's information

Maria Baltzer has an MSc in Social Anthropology from Uppsala University. She has worked primarily with different types of qualitative methods (among others, QCA) in projects concerning culture and health at the Stress Research Institute and at the Swedish Institute of Public Health. At the Stress Research Institute the main studies focus on organizational changes and on work-life balance.

Hugo Westerlund is Professor of Epidemiology at the Stress Research Institute, Stockholm University. Prof. Westerlund focuses on the health consequences of exposures in working life, e.g. organizational changes and labour market exit, particularly retirement. Sickness absence and presenteeism have been the main themes in Prof. Westerlund's research, and he has also studied how managerial leadership practices influence the health of the subordinates. Although mainly a quantitative researcher, Prof. Westerlund also conducts qualitative studies of organizations and their employees.

Mona C Backhans has an MSc in Sociology and is currently a PhD student in Social Medicine at Karolinska Institutet with a focus on gender policy, gender equality and health. As an employee at the National Institute of Public Health, MCB was primarily engaged in studying health consequences of the work environment and labour market status, especially in relation to social inequalities.

Karin Melinder has a Ph D from Karolinska Institutet in Social Medicine. In her dissertation she used QCA regarding cultural aspects of safety in different European countries. She now works at the Swedish Institute of Public Health with regional analyses. Recently she has been the project leader of a Swedish application of the Marmot-review.

## Pre-publication history

The pre-publication history for this paper can be accessed here:

http://www.biomedcentral.com/1471-2458/11/318/prepub

## Supplementary Material

Additional file 1 **Flow chart**. Flow chart describing the selection of informants. Click here for file

Additional file 2 **Informants**. Detailed description of the informants. Click here for file
