# Supplementary material for: Involvement and structure: A qualitative study of organizational change and sickness absence among women in the public sector in Sweden
Source: BMC Public Health. 2011 May 16;11:318. doi: 10.1186/1471-2458-11-318 (PMC3114725; doi:10.1186/1471-2458-11-318)
Supplement: Additional file 1 — Flow chart. Flow chart describing the selection of informants. [file 1471-2458-11-318-S1.DOC]

Jack-knife procedure based on analysis from Westerlund et al 2004

The Swedish population aged 16-64

Labour Force Survey 1989

Stratified random sample 1989

Non-respondents

Labour Force Survey 1991

Stratified random sample 1991

Non-respondents

Labour Force Survey 1993

Stratified random sample 1993

Non-respondents

Labour Force Survey 1995

Stratified random sample 1995

Non-respondents

Labour Force Survey 1997

Stratified random sample 1997

Non-respondents

Labour Force Survey 1999

Stratified random sample 1999

Non-respondents

Work Environ-ment Survey

Non-respondents

Work Environ-ment Survey

Work Environ-ment Survey

Work Environ-ment Survey

Work Environ-ment Survey

Work Environ-ment Survey

Outside of labour force

Non-respondents

Outside of labour force

Non-respondents

Outside of labour force

Non-respondents

Outside of labour force

Non-respondents

Outside of labour force

Non-respondents

Outside of labour force

The ‘RALF’ Database

71,506 individuals

65 years or older in 1999

14,617 (mostly younger) with no data on social class in the 1990 census

27,618 not in employment every November between 1991 and 1996

1,440 who lived outside of Sweden 1997-1999

1,781 who had received unemployment benefit 1997-1999

2,014 with 2 years of 2 different exposures, for mathematical reasons

24,036 persons

14,710 private sector employees

2,778 male public sector employees

6,548 female public sector employees

List where the 6,548 women are ranked according to how much they contributed to the result regarding the association between large-scale expansion and increased risk of long-term sickness absence

List where the 6,548 women are ranked according to how much they contributed to the result regarding the association between moderate expansion and decreased risk of long-term sickness absence

The 200 highest ranked* – Group I

The 200 lowest ranked* – Group III

The 200 highest ranked* – Group II

The 200 lowest ranked* – Group IV

86 non-respondents

74 not interested in interview

7 interviews conducted

31 not selected for interview

1 dead + 1 too ill

75 non-respondents

5 interviews conducted

24 not selected for interview

1 declined

6 interviews conducted

83 non-respondents

3 interviews conducted

40 agreed to be contacted

30 agreed to be contacted

41 agreed to be contacted

29 agreed to be contacted

**A couple of persons were eligible to be part of more than one of the groups below. Those individuals were assigned to the group where they had the most extreme ranking, and were replaced in the other group by the next person in turn.*

95 not interested in interview

89 not interested in interview

70 non-respondents

88 not interested in interview

35 not selected for interview

26 not selected for interview

Baltzer *et al.,* Involvement and structure. *BMC Public Health*, 2011*.* Additional file 1: Flow chart.
