# Supplementary material for: Involvement and structure: A qualitative study of organizational change and sickness absence among women in the public sector in Sweden
Source: BMC Public Health. 2011 May 16;11:318. doi: 10.1186/1471-2458-11-318 (PMC3114725; doi:10.1186/1471-2458-11-318)
Supplement: Additional file 2 — Informants. Detailed description of the informants. [file 1471-2458-11-318-S2.DOC]

Baltzer *et al*., Involvement and structure. *BMC Public Health*, 2011.

Additional file 2: Informants.

The decades when the informants were born

| Pseudonym | Group | Birth decade | Profession |
| --- | --- | --- | --- |
| Monica | I | 1930s | Office worker |
| Mary | I | 1930s | District nurse |
| Anna | I | 1940s | Nursing teacher |
| Sara | I | 1940s | Finance clerk |
| Inga | I | 1950s | Registered nurse |
| Mona | I | 1950s | Childminder |
| Sofia | I | 1950s | Home-help service manager |
| Maud | II | 1930s | Pre-school teacher |
| Birgit | II | 1940s | Social worker |
| Carolina | II | 1940s | Financial secretary |
| Kerstin | II | 1940s | Group leader of a care institution |
| Olga | II | 1940s | Specialized nurse |
| Birgitta | II | 1950s | Social worker |
| Ann-Sofi | III | 1930s | Nurse Assistant |
| Eva | III | 1930s | School assistant |
| Gertrud | III | 1930s | Dental hygienist |
| Maggan | III | 1930s | Personnel manager |
| Gunilla | III | 1950s | Assistant nurse |
| Barbro | IV | 1940s | District nurse |
| Tuulia | IV | 1940s | Office employee, assistant clerk |
| Maria | IV | 1950s | Childminder |
